# Nuclear Deformation During Neutrophil Migration at Sites of Inflammation

**DOI:** 10.3389/fimmu.2018.02680

**Published:** 2018-11-16

**Authors:** Melanie Salvermoser, Daniela Begandt, Ronen Alon, Barbara Walzog

**Affiliations:** ^1^Walter-Brendel-Centre of Experimental Medicine, University Hospital, Planegg-Martinsried, Germany; ^2^Institute of Cardiovascular Physiology and Pathophysiology, Biomedical Center, Planegg-Martinsried, Germany; ^3^Department of Immunology, The Weizmann Institute of Science, Rehovot, Israel

**Keywords:** inflammation, neutrophil, migration, nuclear deformation, myosin1f

## Abstract

Cell migration is indispensable for various biological processes including angiogenesis, wound healing, and immunity. In general, there are two different migration modes described, the mesenchymal migration mode and the amoeboid migration mode. Neutrophils rapidly migrate toward the sites of injury, infection, and inflammation using the amoeboid migration mode which is characterized by cell polarization and a high migration velocity. During site-directed trafficking of neutrophils from the blood stream into the inflamed tissue, neutrophils must first withstand shear stress while migrating on the 2-dimensional endothelial surface. Subsequently, they have to cross different physical barriers during the extravasation process including the squeezing through the compact endothelial monolayer that comprises the blood vessel, the underlining basement membrane and then the 3-dimensional meshwork of extracellular matrix (ECM) proteins in the tissue. Therefore, neutrophils have to rapidly switch between distinct migration modes such as intraluminal crawling, transmigration, and interstitial migration to pass these different confinements and mechanical barriers. The nucleus is the largest and stiffest organelle in every cell and is therefore the key cellular element involved in cellular migration through variable confinements. This review highlights the importance of nuclear deformation during neutrophil crossing of such confinements, with a focus on transendothelial migration and interstitial migration. We discuss the key molecular components involved in the nuclear shape changes that underlie neutrophil motility and squeezing through cellular and ECM barriers. Understanding the precise molecular mechanisms that orchestrate these distinct neutrophil migration modes introduces an opportunity to develop new therapeutic concepts for controlling pathological neutrophil-driven inflammation.

## Introduction

**Figure 1:**
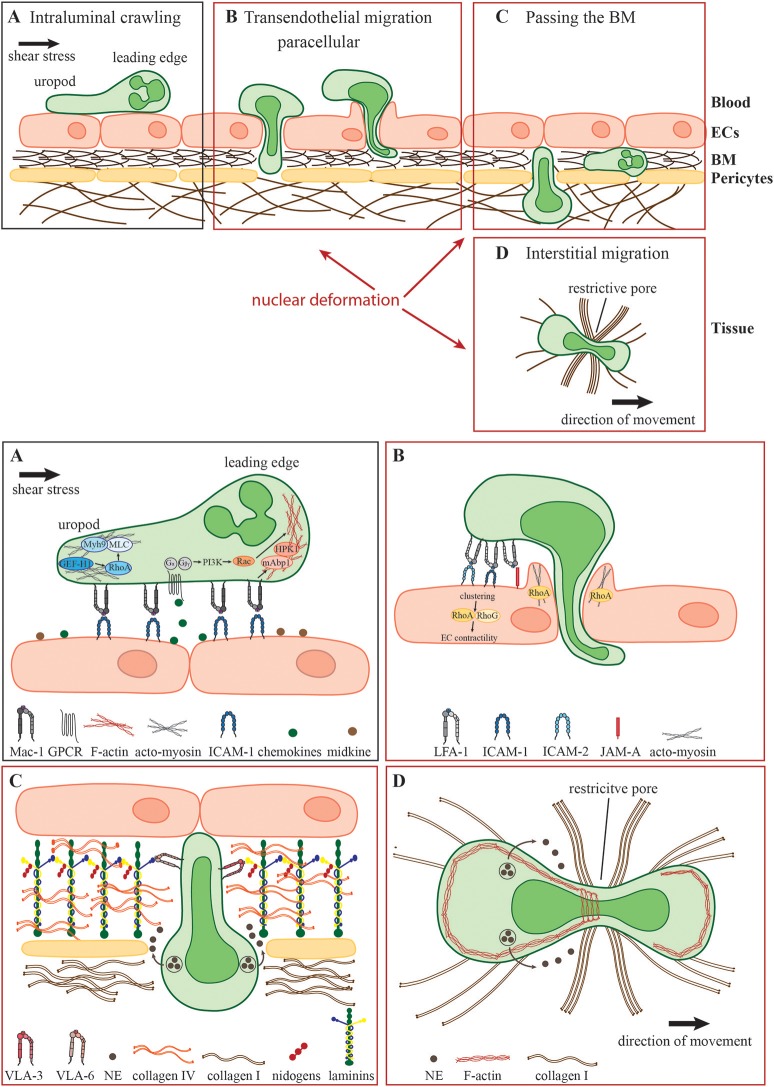
Neutrophil migration in different environmental conditions. During the acute inflammatory response, **(A)** recently arrested neutrophils migrate along the inflamed endothelium (intraluminal crawling) toward potential exit sites. Intraluminal crawling is mainly mediated by the interaction of Mac-1 on neutrophils with ICAM-1 on inflamed ECs. Binding of chemokines and other chemoattractants to their respective GPCRs results in the dissociation of the Gα and Gβγ subunits with subsequent intracellular signaling inducing cell polarization with an F-actin-rich leading edge and an acto-myosin-rich uropod. **(B)** Neutrophils protrude and transmigrate through the endothelial monolayer via the paracellular route. Here, LFA-1- and Mac-1-engagement of endothelial ligands including ICAM-1, ICAM-2, and JAM-A activates endothelial Rho-GTPases e.g., RhoA, RhoG, and NMII leading to EC contractility and plasma leakage restriction. Neutrophils must use their own Rho GTPases to squeeze their nuclei through paracellular endothelial junctions triggering gap formation. **(C)** Following transmigration, neutrophils pass the subjacent basement membrane (BM) and often also crawl on adjacent pericytes embedded in the BM. The main components of the BM are laminins, collagen type IV and nidogens. Neutrophils penetrate this meshwork through LER which can be enlarged by the secretion of elastase (NE) and by nuclear squeezing, both potentially coordinated by the neutrophil integrins VLA-3 and VLA-6 and their interactions with BM collagens and laminins. **(D)** In the inflamed tissue, neutrophils migrate within a 3D collagen I-rich environment toward the site of inflammation (interstitial migration). Here, along with NE secretion, neutrophils deform and push forward their nuclei to pass through restrictive barriers in the meshwork of collagen fibers, most probably by dynamic interactions of their actin cytoskeleton with the nuclear lamina.

Neutrophils are important players in innate immunity as they represent the first immune cells arriving at site of tissue injury or infection. Besides their ability to control local infections, neutrophils are critically involved in tissue remodeling including wound healing, angiogenesis, and tumor metastasis (1–6). During acute inflammation, neutrophils are recruited from the blood stream into the inflamed tissue following a well-defined multi-step recruitment cascade. This cascade is initiated by neutrophil capturing via specific adhesion receptors on inflamed blood vessels, followed by fast and slow rolling, arrest, adhesion strengthening, intraluminal crawling, and protrusion through endothelial junctions in search for exit cues (7). These intravascular events are then followed by neutrophil squeezing through junctions ending in successful transendothelial migration (TEM), followed by abluminal crawling of the neutrophil in between the endothelial layer and its associated pericyte sheet, and interstitial migration to the final destination at the site of inflammation (8). An indispensable prerequisite for efficient neutrophil recruitment is their ability to migrate in different microenvironments. Following 2-dimensional (2D) crawling on the inflamed endothelium in search of potential exit sites, neutrophils protrude and transmigrate through the endothelial monolayer establishing sub-endothelial crawling which involves simultaneous engagement of the endothelial layer and the subjacent basement membrane (BM). Soon thereafter the neutrophil begins to crawl on and navigate in between individual pericytes on its way to the interstitial space where they migrate in a 3D collagen-rich environment toward the site of inflammation (Figure 1) (9–11). During these processes, neutrophil migration is characterized by rapid shape changes underlying polarization into a lamellipodium and a uropod (12, 13). In this review, we describe the diverse environmental conditions which dictate the different migration modes neutrophils employ with a stress on the molecular mechanisms of nuclear deformation events critical for neutrophil squeezing through different cellular (i.e., endothelial and pericytic), and extracellular barriers at sites of inflammation.

## Environmental challenges for neutrophil migration to sites of inflammation

An important characteristic of neutrophils is their high flexibility to adapt their mode of migration rapidly to the environmental conditions. Intraluminal crawling occurs either under low hemodynamic shear stress conditions in postcapillary venules during acute inflammation or under high shear stress conditions in inflamed arteries e.g., during the development of a chronic disease such as atherosclerosis (14). How neutrophils resist shear stress has been reviewed in detail elsewhere (15). Briefly, the process of intraluminal crawling involves specific β_2_ integrin-mediated shear resistant adhesive interactions of neutrophils with endothelial cells (ECs) (Figure 1A). The key integrin ligands on inflamed ECs that enable efficient intravascular neutrophil crawling are ICAMs (16). During this mode of 2D migration β_2_ integrins anchor neutrophils to the adhesive substratum enabling force transmission from the actin cytoskeleton to the environment (17–20). Additional molecules that facilitate intravascular crawling are the cytokine midkine and the serine protease Cathepsin G (CatG) (21, 22). Weckbach et al. demonstrated that the genetic absence of midkine abrogates neutrophil adhesion and extravasation in TNF α-stimulated mouse cremaster muscle venules arguing for a pro-adhesive role of midkine probably by binding to the neutrophil LDL-receptor-related protein-1 (LRP-1) (6, 21, 23). In contrast, CatG displayed by ECs has been found to be exclusively important for neutrophil adhesion to arteries under high flow conditions (22). Integrin-dependent neutrophil adhesion and crawling require the binding of chemokines presented by inflamed blood vessels with respective G-protein-coupled receptors (GPCR) on neutrophils eliciting intracellular signaling that triggers integrin adhesiveness, as well as shape changes and polarization (24). Upon GPCR engagement, primarily CXCR2 (25), the G-protein dissociates into distinct Gαi and Gβγ subunits, which regulate the activity of different molecules such as ion channels, adenylyl cyclase and phosphatidylinositol 3-kinase (PI3K) (26, 27). Activation of the PI3K leads to the recruitment of small guanosine triphosphatases (GTPases) of the Rho family including Rac, Cdc42, and RhoA. Neutrophil GPCRs can also activate these different Rho GTPases via PI3K-independent pathways (28). The appropriate subcellular and spatiotemporal regulation of these signaling molecules mediate cell polarization into an F-actin-rich lamellipodium and a myosin-rich trailing edge—a prerequisite of the amoeboid migration mode (29–31). The non-muscle myosin class II (NMII) protein complex is fundamentally important to maintain cell polarization by linking and translocating F-actin filaments (32, 33). Recently, Zehrer et al. demonstrated the important role of Myh9, the heavy chain of NMIIa in neutrophils for their proper 2D migration (crawling), TEM, and 3D migration. Myh9 was found to be critical for the retraction of the uropod and the consolidation of the leading edge ensuring proper neutrophil polarization and migration (34). Notably, integrin-mediated neutrophil crawling can occur with, against and perpendicular to the direction of blood flow ensuring optimal scanning capacity of the endothelial surface for appropriate extravasation sites (35). Neutrophil crawling to and protrusion through endothelial junctions are regulated by specific cytoskeletal adaptors such as the Rho-GTPase specific guanine exchange factor (GEF) Vav1, the mammalian actin binding protein 1 (mAbp1), the hematopoietic progenitor kinase 1 (HPK1), and GEF-H1 (36–39). It has been shown that CXCL2-stimulated neutrophils use Vav1 for their shear stress-induced perpendicular crawling as the genetic absence of Vav1 results in migration exclusively in the direction of blood flow. Under artificial shear free conditions, the migration behavior of neutrophils is intact in the genetic absence of Vav1 pointing toward the specialized role of Vav1 for Rho activities orchestrating integrin-mediated neutrophil crawling under shear flow (36). The same is true for mAbp1 and its interacting protein HPK1 indicating that these two proteins are additionally required for neutrophil crawling under shear flow (37, 38). Recently, Fine et al. demonstrated that spreading and mechanotactic migration are impaired in the genetic absence of GEF-H1, a specific RhoA GEF (29, 39). These data indicate that neutrophils possess tightly regulated molecular mechanisms that allow their integrin-mediated migration on inflamed vessels under shear stress conditions, a critical checkpoint in their extravasation into inflamed tissues. Once reaching potential exit sites, primarily paracellular endothelial junctions, neutrophils traverse the endothelial monolayer via sequential steps of protrusion through these junction, formation of large pseudopodia in the subendothelial compartments and squeezing of their multi-lobular nuclei through adjacent ECs (Figure 1B). The ECs lining the blood vessel are connected by elaborated endothelial junctions composed of variable tight junctions, adherens junctions and gap junctions (40). In general, neutrophils take almost exclusively the paracellular route for their TEM (41). Neutrophil adhesion triggers also EC signaling events believed to facilitate the disassembly of the endothelial junctions enabling neutrophils to send their protrusions through the endothelial monolayer in search for exit signals, primarily chemokines highly enriched within the endothelial BM (42). Engagements of neutrophil integrins with different endothelial ligands like ICAM-1, ICAM-2, and JAM-A initiates the formation of “docking structures” (43) or “transmigratory cups” (44) consisting of pseudopod-like, F-actin-rich endothelial membrane extensions surrounding the leukocyte (45). Subsequent activation of endothelial Rho-GTPases like RhoA, and RhoG, and NMII triggers EC contractility temporally linked to gap formation (46–48). However, recent works have proposed an alternative mechanism whereby endothelial RhoA and acto-myosin contractility are not required for gap formation by transmigrating neutrophils. Instead, gap formation is dictated by neutrophil protrusion and nucleus squeezing through the paracellular endothelial junctions and at rare instances also through transcellular pores, which generate large displacements of the highly elastic endothelial stress fibers and collapse of thin actin filaments interlaced in between these actin bundles (49, 50). One of these works suggested that RhoA activation in endothelial cells is essential for restricting plasma leakage through the gaps generated by squeezing neutrophils (49). Collectively these studies suggested that neutrophils rather than endothelial cells control their TEM dynamics and that nuclear squeezing determines both the gap size generated by transmigrating leukocytes and the speed of TEM (50).

After successful TEM, neutrophils have to pass the perivascular BM predominantly consisting of laminins (isoform 411 and 511), collagen type IV, heparan sulfate proteoglycans, and nidogens (51–56) as well as embedded pericytes adjacent to the blood vessels (Figure 1C) (57, 58). The venular BM exhibits low-expression regions (LER) of laminins and collagen IV which are enriched between pericytes and are favored exit sites for neutrophils to overcome the BM (59, 60). However, the exact mechanism how neutrophils penetrate the BM is still highly debated. Neutrophils contain specific proteases including matrix metalloproteases and the serine protease neutrophil elastase (NE) and use these proteases to degrade the BM and squeeze through LERs (61, 62). Indeed, elastase-deficient neutrophils can normally cross inflamed endothelium but fail to penetrate the BM (62). In addition, the binding of the neutrophil integrins VLA-3 (α3β1) and VLA-6 (α6β1) to the BM is thought to facilitate neutrophil remodeling of the BM enlargement of LER and interstitial migration at sites of inflammation (59, 63, 64). Interestingly, VLA-3, VLA-6, and NE are located in intracellular vesicles which need to be translocated to the cell surface for efficient neutrophil transmigration through the BM, implicating these integrins as potential scavengers of elastase that restricts its proteolytic activity to BM regions enriched with VLA-3 and VLA-6 binding collagens and laminins (65, 66). Recently, Kurz et al. showed that the mammalian sterile 20-like kinase 1 (Mst1) is critically involved in this unique mobilization of VLA-3, VLA-6, and NE to the neutrophil surface (67). Accordingly, Mst1-deficient neutrophils that successfully extravasate through the venular wall get stuck between the endothelial monolayer and the BM and fail to pass the BM. Neutrophil crossing of the endothelial BM is also tightly associated with neutrophil crawling along venular pericytes (59, 68). This type of 2D migration is also ICAM-1-dependent, and during the onset of inflammation pericytes upregulate this ligand for neutrophil LFA-1 and Mac-1 (68). Whether these neutrophil integrins also rely on stimulatory chemokines co-elevated on inflamed pericytes for crawling, a migration mode that takes place in the absence of shear forces is unknown. It is likely, however, that the GTPase machineries discussed above as critical for β_2_ integrin-mediated neutrophil crawling on inflamed endothelial cells under shear flow- are not identical to those involved in neutrophil crawling on inflamed pericytes.

In the inflamed tissue, neutrophils migrate in 3D collagen-rich environments toward their final destinations at the site of inflammation (Figure 1D). Of note, the microenvironment in which the neutrophils migrate differs both mechanically and biochemically between different organs (69). However, the extracellular matrix as the non-cellular component of all tissues consists predominantly of type I collagen, elastin, proteoglycans, and non-collageneous glycoproteins (70). Here, type I collagen assembles into mechanically stable fibrils providing physical stability of the connective tissue (71). *In vivo* this fibrillary collagen meshwork exhibits interfibrillar spaces ranging from 2 to 30 μm as shown for mouse cremaster tissue (71, 72). Neutrophils migrate within this confined tissue in a low-adhesive and largely β_2_ integrin-independent manner. Furthermore, integrin-deficient as well as talin-deficient neutrophils show intact migration in 3D environments compared to control cells, ruling out contributions from either β_1_ and β_3_ integrins to this mode of neutrophil motility (17, 73). These data indicate that the traction forces needed for successful 3D migration are transmitted to the environment without integrin-dependent anchoring of the cell to the surface, the prevalent mechanism for neutrophil migration in 2D environments (17, 74). However, the exact mechanism how neutrophils translate their intracellular actomyosin-driven forces to the traction forces critical for their locomotion inside various collagenous 3D environments is still not entirely understood.

In order to study the underlying mechanism experimentally, 3D collagen gels are widely employed. These gels mimic different meshwork architectures with different pore sizes, dependent on the collagen concentration. A collagen concentration of 1.5 mg/mL yields a low-density meshwork with pore cross sections of 10–12 μm^2^ and a high-density collagen matrix with a collagen concentration of 3.0 mg/mL exhibits pore cross sections ranging between 2 and 3 μm^2^ (17, 72). As the exact structure of collagen gels cannot be experimentally controlled, various microchannels were recently developed to closely mimic parameters including pore sizes and micro-geometry to improve the analysis of interstitial migration (75, 76). During migration in such confined 3D environments, neutrophils need to pass physical restrictions much smaller than their nucleus similar to the situation in the tissue or 3D collagen gels. Nevertheless, while microchannels are rigid, dense 3D collagen polymers are not only more elastic but can be also locally degraded by neutrophil proteases. Thus, neutrophil passage through microchannels and collagen barriers involve similar but not identical requirements of nucleus deformation.

## Molecular mechanisms of nuclear deformation

**Figure 2:**
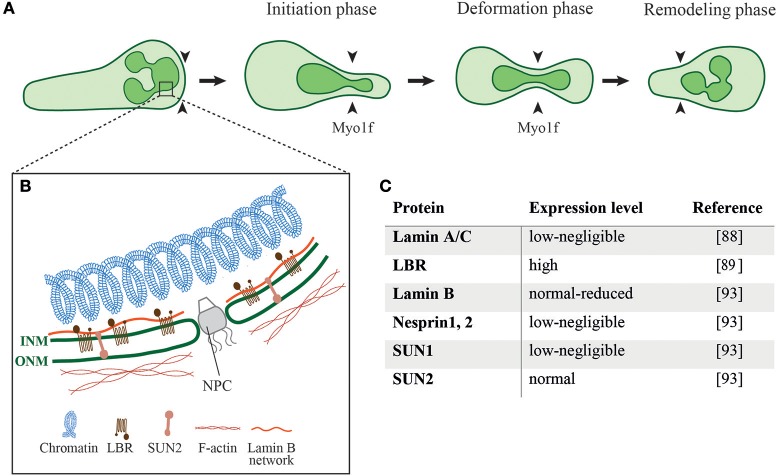
Different phases of nuclear deformation and structural components of the neutrophil nucleus. **(A)** While neutrophils squeeze through restrictive barriers (indicated by black arrowheads) smaller than their nucleus, the individual nuclear lobes undergo different phases of deformation. During the initiation phase neutrophils use one of its preexistent lobes to penetrate the barrier. This is followed by pushing, deformation, and elongation of the lobe and its neighbor lobes. Myo1f is critically required for nuclear pushing and deformation during this squeezing process. **(B)** Schematics of the structural proteins that regulate the shape and mechanical properties of a neutrophil nucleus as well as its crosstalk with the neutrophil cytoskeleton. ONM, outer nuclear membrane; INM, inner nuclear membrane; NPC, nuclear pore complex; LBR, Lamin B receptor; **(C)** Expression levels of different nuclear proteins in neutrophils.

During cell migration through different mechanical constrictions the dynamic interaction of the nucleus with the actin cytoskeleton is required to ensure proper positioning of the nucleus and nuclear deformation to successfully squeeze the cell through these constrictions (77). Indeed, nucleus deformation is the rate-limiting step for cells to pass through different constrictions smaller than the nucleus (78–81). The neutrophil nucleus is composed of 2–6 nuclear lobes with a diameter of 2 μm connected by a segment with a size of ~ 0.5 μm (82, 83). Nuclear deformation follows three different phases while the cell squeezes through physical barriers, namely the initiation phase, the deformation phase and the remodeling phase (Figure 2A) (78, 84). When the cell reaches the constriction, the nucleus is the first organelle pushing against the constriction (50, 84). During the deformation phase the nucleus elongates into an hour-glass shaped nuclear morphology while squeezing through the constriction. After passing the constriction, the rear of the nucleus pushes forward to refold into its original spherical morphology. The nucleus is mechanically stabilized by a thin, elastic shell encoded by three genes, LMNA, encoding lamins A/C, and LMNB1 and LMNB2, encoding lamin B1 and lamin B2, respectively (85). The rigidity of the nuclei is determined by the relative levels of their A and B lamins (86, 87). The nuclear lamina of neutrophils is much softer than the lamina of most tissue resident cells due to their negligible content of lamin A/C (Figures 2B,C) (88). The neutrophil nucleus is further adjusted for rapid squeezing through small confinements by its unique multi-lobular shape. Expression of a lamin B receptor (LBR) on the inner nuclear membrane is critical for this multi-lobular shape (Figures 2B,C) (89). Interestingly, interference with the multi-lobular shape of the nucleus by LBR knockdown keeping lamin A content low bears minimal effects on nuclear squeezing via rigid pores (90). Notably, bone marrow neutrophil precursors regulate both their nuclear shape and lamin A/C content during maturation. The nuclei of immature neutrophils are stiff and circular as they express higher levels of lamin A/C and lack LBR. Upon full maturation neutrophils adapt their nuclear shape and rigidity to optimize their squeezing through bone marrow sinusoids (89). Similarly, naïve T cells temporally upregulate their lamin A/C expression during TCR activation and remain stationary until they downregulate lamin A/C expression and regain nuclear deformability as they become migratory (91). Thus, nuclear shape and deformability are adapted to the squeezing needs of particular cells.

In contrast to epithelial and mesenchymal cells, which keep their stiff nuclei at their rear, motile leukocytes readily translocate their nuclei to their pseudopodia and do so irrespectively of barrier rigidity (50). In a recent study on granulocyte-like differentiated HL-60 cells, we found that this property of the neutrophil nucleus is conserved and is independent of the barrier rigidity the neutrophil is squeezed through. Nevertheless, when the stiffness of the nucleus was elevated by overexpression of lamin A, and when the neutrophil was embedded in a dense collagen matrix the nucleus could no longer translocate to the neutrophil pseudopodia (92). Thus, the exceptional ability of neutrophils to squeeze through mechanically rigid barriers such as collagen-rich interstitial spaces, or the BMs of blood vessels and epithelial barriers likely depends on both low lamin A content, nuclear lamina deformability, and high LBR expression (90). These requirements are dispensable, however, for neutrophil squeezing through the much softer endothelial junctions and consequently for TEM, because of the higher elasticity of the endothelial cytoskeleton than the elasticity of individual collagen fibers within collagen-rich interstitial spaces and the BMs of blood vessels and epithelial barriers.

The nuclear translocation to the neutrophil's pseudopodia, a shared feature among all motile leukocytes which appears to facilitate their squeezing may be regulated by specific interactions of the nuclear cytoskeleton (nucleoskeleton) and the perinuclear actin filaments. The neutrophil nucleoskeleton is deficient of several linker of the nucleoskeleton and cytoskeleton (LINC) complex proteins, including nesprin1, 2, and SUN1 (Figures 2B,C) (93) which are implicated in force transmission in adherent cells (94). Mature neutrophils are possibly devoid of these nuclear-cytoskeletal interactions as part of their highly motile nature and preference of chemotactic cues over integrin-dependent adhesions specialized to transduce forces to the nucleus (95). The nuclei of neutrophils can be also pushed to the leading edge by actomyosin machineries that orchestrate nuclear positioning and squeezing and bridge the neutrophil's uropod with the microtubule-organizing center (MTOC) at the back of the squeezed nucleus (96).

In addition to the unique shape and deformability of the neutrophil nucleus, neutrophil migration to sites of inflammation critically depends on the unconventional class I myosin Myosin 1f (Myo1f), found to facilitate nuclear deformation (84). This recent work suggests that the deformation of the nucleus is almost completely absent in Myo1f-deficient neutrophils compared to control cells resulting in diminished *in vitro* neutrophil migration within 3D collagen gels and impaired *in vivo* trafficking toward sites of lesions. Whereas, class II myosins are involved in the generation of contractility forces, class I myosins exist as monomers and link membranes to the actin cytoskeleton (97) potentially implicating these myosins in nuclear deformation critical for neutrophil squeezing. How precisely this unique myosin communicates with the nucleus and its closely associated microtubules remains an open question for future investigations.

## Conclusion

Neutrophil migration to sites of inflammation is indispensable for innate immunity as neutrophils are the predominant immune cells combating pathogens. Efficient neutrophil migration critically relies on the exceptionally dynamic deformation of the nucleus of neutrophils. Accordingly, neutrophils obtained from mice lacking LBR expression show hyposegmentation of the nucleus associated with a decreased nuclear deformability and impaired neutrophil responses (98). The same is true for patients suffering from Pelger-Huet anomaly (PHA), a mutation in the human LBR leading to hyposegmentation of the neutrophil nucleus (99). Thus, impaired nuclear deformability can hamper neutrophil migration and function in inflammation. The improvement of our current knowledge of the molecular mechanisms underlying nuclear deformation events critical for neutrophil crossing through distinct mechanical barriers may therefore help to identify novel therapeutic targets for the treatment of neutrophil-driven acute and chronic inflammatory pathologies as well as for the manipulation of neutrophil crosstalks with tumor cells.

## Author contributions

All authors listed have made a substantial, direct and intellectual contribution to the work, and approved it for publication.

### Conflict of interest statement

The authors declare that the research was conducted in the absence of any commercial or financial relationships that could be construed as a potential conflict of interest.
